# Overcontrolled, undercontrolled, and resilient personality styles among patients with eating disorders

**DOI:** 10.1186/s40337-021-00400-0

**Published:** 2021-04-16

**Authors:** Martina Isaksson, Ata Ghaderi, Martina Wolf-Arehult, Mia Ramklint

**Affiliations:** 1 Department of Neuroscience, Psychiatry, Uppsala University, Entrance 10, Floor 3B, SE-751 85 Uppsala, Sweden; 2 Division of Psychology, Department of Clinical Neuroscience, Karolinska Institutet, SE-171 77 Stockholm, Sweden; 3 Stockholm Centre for Eating Disorders, Stockholm Health Care Services, Stockholm County Council, SE-171 77 Stockholm, Sweden; 4 Psychiatry Northwest, Region Stockholm, Clinical Management, PO Box 98, SE-191 22 Sollentuna, Sweden

**Keywords:** Eating disorders, Anorexia, Bulimia, Overcontrol, Undercontrol, Resilience

## Abstract

**Background:**

Personality has been suggested to be an important factor in understanding onset, maintenance, and recovery from eating disorders (ED). The objective of the current study was to evaluate personality style in different ED diagnostic groups as classified in the Diagnostic and Statistical Manual of Mental Disorders, Fifth edition (DSM-5).

**Methods:**

The overcontrolled, undercontrolled, and resilient personality styles were compared in four groups of patients with EDs: anorexia nervosa restricting (ANr) (*n* = 34), anorexia nervosa binge eating/purging (ANbp) (*n* = 31), atypical anorexia nervosa (AAN) (*n* = 29), and bulimia nervosa (BN) (*n* = 76). These groups were compared with a group of patients with borderline personality disorder (BPD) (*n* = 108), and a non-clinical group (NC) (*n* = 444). Patient data were collected at two outpatient clinics in Uppsala, Sweden. NC control data were collected through convenience sampling. Participants filled out questionnaires assessing personality style.

**Results:**

The main findings were more pronounced overcontrol reported by the ANr and AAN groups compared with the BN, BPD, and NC groups, and no significant difference in resilience between the ED and the NC groups. Considerable variability of over- and undercontrol was also found within each group.

**Conclusions:**

The results replicate previous findings when EDs are classified according to current diagnostic criteria (DSM-5). Taking personality styles into account may improve our understanding of certain characteristics in EDs, such as social deficits and rigidity that are attributed to poor treatment outcome.

## Plain English summary

To increase our understanding of eating disorders (ED), we need to know more about risk factors for developing as well as maintaining EDs. In this study, we evaluated if overcontrol (characterized by rigidity, perfectionism, and inhibition of feelings), is more common in certain eating disorder difficulties compared to undercontrol (characterized by impulsivity and immediate expression of feelings). We also evaluated if the groups differed when contrasted to a group in the community and to patients with borderline personality disorder (BPD; characterized by excessive undercontrol). Finally, we evaluated if resilience, such as being able to flexibly adapt to changing demands in the environment, differed between those with EDs and the community group. Our results showed that restricting and atypical anorexia nervosa had the highest levels of overcontrol, that BPD had the highest level of undercontrol, and that bulimia and the community sample were in between. Further, there were no differences in resilience between the ED group and the community sample. Our findings may help us understand more about factors that are contributing to poor treatment outcome in anorexia, such as rigidity and social difficulties.

## Introduction

Researchers studying the etiology of eating disorders (ED) have suggested that personality is an important factor in the understanding of EDs, likely to affect how symptoms are exhibited and maintained [1, 2]. Knowledge of personality characteristics contributes to better prediction of level of functioning, clinical course, and treatment outcome – beyond specific ED diagnoses [1, 3, 4].

Several studies have identified three personality subtypes in the ED field [3–6]: one constricted overcontrolled, one emotionally dysregulated undercontrolled, and one high-functioning perfectionistic. Most commonly, personality constructs related to overcontrol or high self-control such as introversion and constraint have been related to anorexia nervosa (AN) and restricting ED subtypes, whereas personality constructs related to undercontrol and low self-control such as impulsiveness have been related to bulimia nervosa (BN) or binge eating/purging ED subtypes [1, 5, 7]. The high-functioning subtype has been found to be more diverse, yet somewhat overrepresented in BN [4]. In a recent, systematic, review by Farstad and von Ranson [1], it was highlighted that almost all research on personality in EDs had been done based on outdated diagnostic criteria from DSM-IV. Also, findings on distinctions between different ED groups have been limited [1], and some have not been able to replicate e.g., overcontrolled and high-functioning personality subgroups in AN [1, 8]. Further, when studying personality constructs in the ED group, subthreshold diagnoses such as atypical AN (AAN; i.e., patients with significant weight loss, but whose weight is still within or above normal range), have rarely been included [1]. Thus, the field is in need of further evaluation and replication.

A personality theory with specific emphasis on emotional control, first developed by Block and Block [9], have identified three recurrent personality styles in different populations [10–12]: the overcontrolled (characterized by high self-control, inhibitory control, and persistence, and low impulsiveness and emotional expression), the undercontrolled (characterized by high levels of impulsivity and emotional expression, with low levels of self-control and inhibition), and the resilient (characterized by an ability to modify the level of control to ever changing demands in the environment) [9, 12–14]. These styles resembles the personality constructs previously identified among EDs, but differs e.g., in terms of the definition of high functioning versus resilience. To our knowledge, the occurrence of resilience in ED groups has only been evaluated by Tsigkaropoulou and colleagues [15]. They found significant differences in resilience between a non-clinical (NC) group and the ED group, but not between ED subgroups.

In sum, identification of personality subtypes has shown clinical utility in predicting e.g., treatment outcome [3], and it has been suggested that identification of personality may be one way to individualize the interventions to improve treatment effectiveness [2]. However, knowing what differs and what is shared between diagnoses, might also facilitate the possibility of adapting the treatments on a group level, to better fit the more common personality style identified in each group.

The aim of the current study was to investigate personality style in restricting AN (ANr), binge eating/purging AN (ANbp), AAN, and BN, classified according to DSM-5. These were to be compared with a group with borderline personality disorder (BPD), expected to be undercontrolled, and a NC group, expected to be high in resilience and intermediate in over−/undercontrol. Replicating findings of personality styles in different ED according to current diagnostic criteria is important, especially as the current classification is more inclusive, which may reduce the somewhat limited distinctions in personality that has previously been identified across diagnoses [1]. Also, including the AAN group provides information about an ED group that is commonly left out [1]. Moreover, by contrasting the ED groups to a NC group and a relevant clinical group (i.e., BPD), the specific characteristics of the ED group in terms of personality will be easier to contextualize. In the current study, we hypothesized: 1) A difference in over- and undercontrol between ED groups, with higher levels of overcontrol in the ANr compared to the BN group, 2) higher levels of overcontrol in the ANr group in comparison with the NC and BPD group, and 3) lower levels of resilience in the ED groups when compared with the NC group.

## Materials and methods

### Procedure

Data were collected at two outpatient clinics, the ED clinic and the BPD clinic, at Uppsala University Hospital. ED patients were recruited between October 2014 and December 2019. The ED clinic treats patients with AN, atypical AN, moderate and severe BN, and other EDs in cases with complex comorbidity or suicidality. Patients with binge eating disorder (BED) and obesity are referred to the obesity unit at the medical clinic, whereas less severe EDs such as subclinical BN are referred to primary care or private practices, and could therefore not be included in the study.

Patients underwent a diagnostic evaluation, including the semi-structured Eating Disorder Examination interview (EDE) [16]. Fifteen psychologists were trained in performing the interview by an expert and co-rated six filmed interviews performed by the expert. All assessors showed complete agreement in all ratings with a prevalence and bias adjusted kappa (PABAK) [17] of 1.0. Diagnoses were initially based on the Diagnostic and Statistical Manual of Mental Disorders 4th ed., Text Revision (DSM-IV-TR) [18], but later recoded based on the DSM-5. Hence, patients not diagnosed with AN under DSM-IV-TR, due to higher weight (17.5 kg/m^2^ < BMI < 18.5 kg/m^2^) and no amenorrhea, were included in AN. Patients fulfilling all criteria from AN except having weight within or above normal, i.e. BMI ≥ 18.5, but with significant weight loss, were included in AAN. Those with a lower frequency of binge eating and compensation (both at least once per week, in contrast to twice per week in previous versions) were included in BN. If a patient was not weighed at the clinic, an approximation of the weight was estimated to enable accurate diagnostics; this was done in one case.

EDs were grouped so that patients diagnosed with AN *without* episodes of binge eating or purging were included in the ANr group, patients diagnosed with AN *with* episodes of binge eating or purging in the ANbp group, patients diagnosed with AAN in the AAN group, and patients diagnosed with BN in the BN group.

Patients with BPD were recruited at the BPD clinic between October 2014 and November 2017. Diagnosis was based on the Structured Clinical Interview for DSM-IV Axis II Personality Disorders, general and borderline criteria (SCID-II) [19], performed by psychologists. All psychologists were trained by an expert in performing the interview, with co-ratings showing a kappa (PABAK) ranging from .6–1.0 between assessors. No recoding was necessary for the BPD group, as diagnostic criteria are the same in both DSM versions.

Patients were informed about the study through verbal and written information at their first meeting during the psychological assessment phase. Participants signed an informed consent and filled out the questionnaires. Weight and height were measured at the clinic.

The NC sample was recruited between October 2014 and June 2016 through information in classes at Uppsala University and advertisement on the web and in social media. Interested participants received more information and a link to the questionnaires online. Participants received two scratch cards or one movie ticket, or could donate the corresponding amount to a charity of their choice.

### Participants

**Fig. 1:**
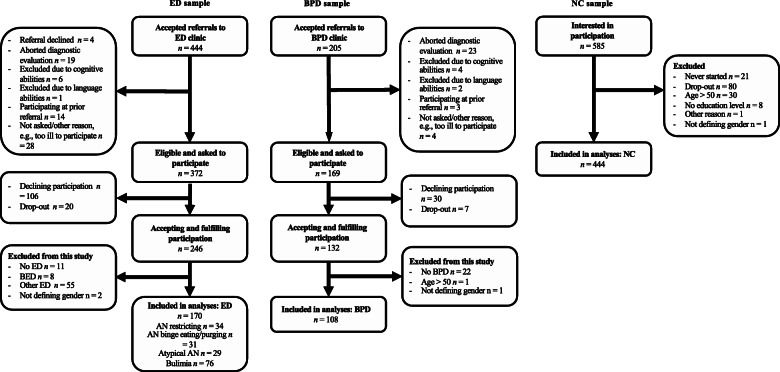
Flowchart of participant recruitment

In total, 170 ED patients (ANr: *n* = 34, ANbp: *n* = 31, AAN = 29, BN: *n* = 76), 108 BPD patients, and 444 NC individuals participated in the study. Inclusion criterion for the NC sample was minimum age of 18 years. For the clinical sample, inclusion was restricted to patients represented at the ED and BPD clinics. Inclusion criteria for patients were 1) minimum age 18 years, and 2) being diagnosed with AN, AAN, BN, or BPD. Excluded were patients 1) already participating in the study at a prior referral, 2) too ill to participate, 3) with insufficient cognitive abilities or knowledge in Swedish 4) with BED (*n* = 8), and 5) with other EDs (*n* = 55), e.g., BN with lower frequency or unspecified feeding or eating disorder. Because all ED patients were 50 years or younger, all participants over 50 years of age (*n* = 31) were excluded from the NC and BPD comparison groups. Subjects not defining an education level (*n* = 8) or not defining gender (*n* = 4) were also excluded from the comparison groups. Recruitment and demographic characteristics are presented in Fig. 1 and Table 1.

**Table 1:** Demographic characteristics of study participants

	ANr	ANbp	AAN	BN	BPD	NC
	(***n*** = 34)	(***n*** = 31)	(***n*** = 29)	(***n*** = 76)	(***n*** = 108)	(***n*** = 444)
**Mean age (SD)**	24.2 (5.1)	24.5 (6.8)	22.7 (3.7)	27.3 (7.8)	26.5 (6.4)	28.7 (8.2)
**Mean BMI (SD)**^**a**^	16.7 (1.2)	17.3 (1.0)	20.0 (1.8)	24.7 (4.7)	–	–
**Gender**						
Female	31 (91.2%)	30 (96.8%)	27 (93.1%)	74 (97.4%)	96 (88.9%)	340 (76.6%)
Male	3 (8.8%)	1 (3.2%)	2 (6.9%)	2 (2.6%)	12 (11.1%)	104 (23.4%)
**Marital status**^**b**^						
Married or in relationship	7 (21.2%)	9 (30.0%)	12 (41.4%)	26 (34.2%)	56 (51.9%)	270 (60.8%)
Single	24 (72.7%)	19 (63.3%)	16 (55.2%)	46 (60.5%)	51 (47.2%)	163 (36.7%)
Other (e.g., living with parents)	2 (6.1%)	2 (6.7%)	1 (3.4%)	4 (5.3%)	1 (0.9%)	11 (2.5%)
**Highest level of education**						
Elementary school	5 (15.2%)	7 (22.6%)	4 (13.8%)	9 (11.8%)	16 (14.8%)	14 (3.2%)
High school	17 (51.5%)	14 (45.2%)	14 (48.3%)	36 (47.4%)	68 (63.0%)	152 (34.2%)
Higher education	11 (33.3%)	10 (32.3%)	11 (37.9%)	31 (40.8%)	24 (22.2%)	278 (62.6%)
**Main occupation**^**c**^						
Paid work	8 (25.0%)	9 (29.0%)	7 (25.0%)	33 (44.0%)	36 (33.6%)	189 (42.6%)
Student	16 (50.0%)	17 (54.8%)	11 (39.3%)	27 (36.0%)	21 (19.6%)	197 (44.4%)
Unemployed	2 (6.3%)	0 (0.0%)	2 (7.1%)	0 (0.0%)	6 (5.6%)	20 (4.5%)
Sick leave	5 (15.6%)	4 (12.9%)	8 (28.6%)	14 (18.7%)	41 (38.3%)	17 (3.8%)
Other (e.g., housewife, internship, parental leave)	1 (3.1%)	1 (3.2%)	0 (0.0%)	1 (1.3%)	3 (2.8%)	21 (4.7%)

*ANr* Anorexia nervosa restricting, *ANbp* Anorexia nervosa binge eating/purging, *AAN* atypical anorexia nervosa, *BPD* borderline personality disorder, *NC* non-clinical

^a^One patient with AAN was missing BMI

^b^one patient with ANr and one with ANbp were missing marital status

^c^ two patients with ANr, one with AAN, one with BN, and one with BPD were missing main occupation

### The instruments

#### The Ego Undercontrol-13 and Ego resilience scales

The Ego Undercontrol (EUC) scale is originally a 37-item self-report questionnaire [9] designed to assess over- and undercontrolled personality styles. Since the original scale has shown insufficient psychometric properties, a short version (the EUC-13) was developed and evaluated in a Swedish study, showing improved psychometrics [20]. The short version had not been developed at the beginning of the study. Therefore, items from the EUC-13 were extracted and a sum was calculated for the EUC-13 for subjects who completed the original EUC. The Ego Resilience (ER) scale is a 14-item questionnaire [9, 21] designed to assess the ability to adapt the level of control based on the circumstances.

Both scales have shown acceptable to high internal consistency (EUC-13: α = .71; ER: α = .84) and test-retest reliability (EUC-13: *r* = .86, *p* < .001; ER *r* = .85, *p* < .001), and acceptable factor structure and validity [20]. Items are reported on a four-point Likert scale ranging from “Disagree very strongly” (1) to “Agree very strongly” (4). For the EUC-13, low scores indicate high levels of overcontrol, and high scores indicate high levels of undercontrol. High scores on the ER indicate high adaptive control. In the present study, internal consistency was .75 for the EUC-13 and .81 for the ER.

### Data analysis

Power analyses were performed to estimate a desired sample size. To detect small changes, the recommended number of participants in each group were estimated at *n* = 35 (given an eta effect size of *η*^*2*^ *=* .25, power = .80, α = .05). However, because of the low prevalence of AN, and because the AN group was divided in to three separate groups, we needed to consider limitations in achieving large samples. Thus, sample size was a bit smaller for some groups, which may imply that it was not possible detect small differences in terms of significance. Moderate to large differences could, however, be detected, and measures of effect are presented to enable interpretation of group differences beyond the issue of significance.

**Fig. 2:**
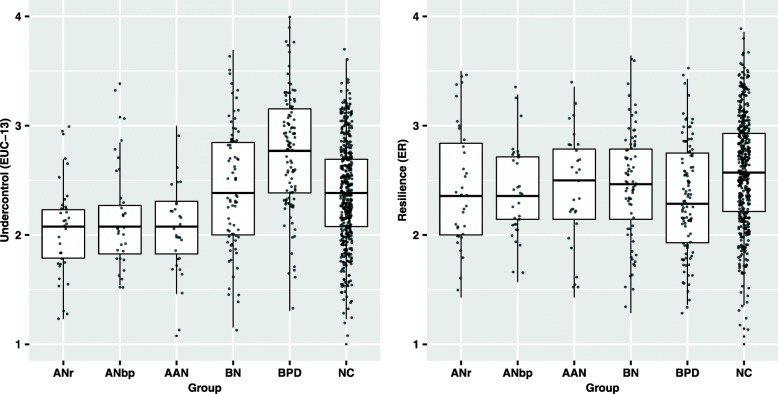
Boxplot and jittered scatterplot of overcontrol, undercontrol, and resilience. *Note.* Group differences on Ego Undercontrol scale – 13 (EUC-13) and Ego Resilience scale (ER) are illustrated for anorexia nervosa restricting (ANr), AN binge eating/purging (ANbp), atypical AN (AAN), bulimia nervosa (BN), borderline personality disorder (BPD), and non-clinical (NC)

Normal distribution was checked with skewness ratio (skewness/SE (skewness)). All measures were below the recommended value of 2.0, supporting the use of linear models. Missing values were rare (< 0.1% of participants), and were imputed with the mean of that subscale. In case of more than one missing value per subscale or two missing for the whole scale, that person was omitted from the analysis. Group differences of overcontrol and resilience were analyzed using a generalized linear model (GZLM). A GLZM is preferred to a general linear model (GLM, e.g., ANOVA) when group sizes are unequal, as it allows specification of a model in which the outcome variable follows different kinds of distributions. We applied two separate crude analyses in the GLZM with the diagnostic group as the independent variable. Overcontrol/undercontrol (measured with the EUC-13) was used as the dependent variable in one analysis and resilience (measured with the ER) in the other. Next, we performed adjusted analyses to see if results were stable when adding gender, age, marital status, and level of education as covariates to the model. Both crude and adjusted results are presented in Table 2. Due to multiple comparisons, we used the Bonferroni post hoc test to adjust the significance level. Because only small differences were seen when adjusting for covariates, crude results were used for all succeeding analyses. To test if the extraction of items from EUC 37 affected the outcome, we performed additional analyses when groups were separated based on what version of the EUC they conducted (the EUC 37 or the EUC-13). No significant differences were identified. Effect sizes were estimated with Cohen’s *d*, with SD derived from the standard error and pooled to correct for differences in sample size. To illustrate the spread of over- and undercontrol, group medians with dispersion were visualized for the EUC-13 and the ER (Fig. 2). Analyses were performed using IBM SPSS Statistics version 25 and R version 3.5.1.

**Table 2:** Differences between eating disorder and comparison groups on overcontrol/undercontrol and resilience

		ANr (***n*** = 34) Mean (SE)	ANbp (***n*** = 31) Mean (SE)	AAN (***n*** = 29) Mean (SE)	BN (***n*** = 76) Mean (SE)	BPD (***n*** = 108) Mean (SE)	NC (***n*** = 444) Mean (SE)	Bonferroni post hoc
**EUC-13**	Crude	2.05 (0.08)	2.14 (0.09)	2.05 (0.09)	2.43 (0.06)	2.76 (0.05)	2.38 (0.02)	ANr < BN**, ANr < BPD***, ANr < NC** ANbp < BPD*** AAN < BN**, AAN < BPD***, AAN < NC**, BN < BPD***, BPD > NC***
	Adjusted	2.08 (0.09)	2.17 (0.09)	2.11 (0.10)	2.47 (0.07)	2.79 (0.06)	2.42 (0.05)	ANr < BN**, ANr < BPD***, ANr < NC**, ANbp < BPD***, AAN < BN*, AAN < BPD***, AAN < NC*, BN < BPD***, BPD > NC***
**ER**	Crude	2.45 (0.09)	2.40 (0.10)	2.44 (0.09)	2.47 (0.06)	2.33 (0.05)	2.58 (0.02)	BPD < NC***
	Adjusted	2.50 (0.09)	2.46 (0.10)	2.47 (0.10)	2.52 (0.07)	2.41 (0.06)	2.60 (0.05)	BPD < NC**

Measures of estimated means (crude/unadjusted) and estimated marginal means (adjusted for covariates) with standard errors (SEs) *for the Ego Undercontrol scale – 13 (EUC-13) and the Ego Resiliency scale (ER).* Significant differences between groups are calculated using the Bonferroni post hoc test, adjusting for multiple comparisons, * *p* < 0.05, ** *p* < 0.01, *** *p* < 0.001

*ANr* anorexia nervosa restricting, *ANbp* AN binge eating/purging, *AAN* atypical AN, *BN* bulimia nervosa (BN), *BPD* borderline personality disorder (BPD), *NC* non-clinical

## Results

### Group differences in personality style

Distribution and dispersion are presented in Fig. 2; unadjusted and adjusted group means with standard errors are presented in Table 2. All significant effects in the crude model were stable when adjusted for relevant covariates.

### Measures of effect in personality style

Investigation of the differences in over- and undercontrol between ED groups showed that the ANr reported higher levels of overcontrol than the BN (*d* = 0.75), as hypothesized. The AAN also reported higher levels of overcontrol than the BN (*d* = 0.74). No other differences were identified between the ED groups in terms of overcontrol. Further, the magnitudes of effects were generally small to moderate, as reflected in the corresponding effect sizes: ANr vs. ANbp (*d* = 0.19), ANr vs. AAN (*d* = 0.00), ANbp vs. AAN (*d* = 0.18), or ANbp vs. BN (*d* = 0.56).

When over- and undercontrol was contrasted to the comparison groups, the ANr group reported significantly higher levels of overcontrol than the BPD group (*d* = 1.40) and the NC group (*d* = 0.78), as hypothesized. All other ED groups also showed higher levels of overcontrol when compared with the BPD groups: BPD vs. ANbp (*d* = 1.20), BPD vs. AAN (*d* = 1.39), and BPD vs. BN (*d* = 0.63). The AAN group showed significantly higher levels of overcontrol than the NC group (*d* = 0.78), while the differences between the NC group and the other ED groups were non-significant: NC vs. ANbp (*d* = 0.56), NC vs. BN (*d* = 0.11).

In contrast to our hypotheses, there were no significant differences between the NC group and any of the ED groups regarding resilience: ANr vs. NC (*d* = 0.30); ANbp vs. NC (*d* = 0.42), AAN vs. NC (*d* = 0.33), and BN vs. NC (*d* = 0.25).

## Discussion

The main findings were a higher level of overcontrol in the ANr and ANN groups compared with the BN, BPD, and NC groups, with medium to large effect sizes. The ANr and AAN groups showed the highest levels of overcontrol, the BPD group showed the highest level of undercontrol, and the BN and NC groups were at the same intermediate level. No significant difference in overcontrol was found when comparing the ANbp group with other ED groups or the NC group. This was, however, probably an effect of the relatively small *n* in the ANbp group, and effect sized provides information of at least small differences between both the ANbp and the BN, and the ANbp and the NC groups – with higher overcontrol in ANbp. Lastly, there were no significant group differences of resilience between the ED and the NC groups.

Our results add strength to the previously identified patterns of over- and undercontrolled personality styles [13, 22], supporting their occurrence in different EDs [3–6, 23]. As we included a BPD comparison group, we were able to conclude that the pattern displayed by the BN group more closely resembled that of the BPD undercontrolled group than did the patterns of any of the other ED groups assessed in the study. An interesting finding, adding information to this field of research, is that the AAN group showed large similarities to the ANr group with regard to levels of overcontrol.

Resilience has previously been associated with well-being and functional control, which is often seen in NC samples and in less severe psychiatric disorders [6, 22]. Hence, as identified in one previous study [15] the NC group was expected to report higher level of resilience, compared with ED groups. Our results did not confirm this expectation. Lack of significant differences might be partly due to low rating of resilience in the NC group. Their ratings were in fact lower than the University students from the original evaluation of the scale [22]. It is also worth emphasizing that the occurrence of a separate resilient high functioning/perfectionistic group, previously identified within EDs [4], was not investigated in this study. Hence, because the high-functioning individuals were included in the over- or undercontrolled groups, resilience ratings were supposedly higher than if they would have been represented by a separate high-functioning group.

### Implications

The present study offers an important contribution to research on EDs, as previous research could be replicated and refined with a thorough diagnostic assessment procedure (i.e., a semi-structured interview) and diagnoses based on DSM-5. Our results, together with previous findings, have several implications. For example, some researchers have suggested that personality should be taken into consideration when planning treatment [2, 14]. Fairburn’s Enhanced Cognitive-Behavioral Therapy for ED (CBT-E) [24] includes ways of individualizing treatment to address personality traits, such as perfectionism. Also, impulsivity could be added as a treatment target for BN patients with an undercontrolled personality style [25], whereas rigidity could be targeted for AN patients with an overcontrolled personality style [26].

Results in the present study also showed a high variability of personality style within each ED group, as illustrated by the individual ratings in the scatterplot. Thus, the relationship between different ED subtypes and personality styles may be more complex than simply describing individuals with restrictive AN as overcontrolled and those with BN as undercontrolled [4, 23]. Heterogeneity within the AN and BN subgroups, and frequent migration between diagnoses [2, 27], highlights the need for improved precision in ED assessments. Other measures, improving prognostic accuracy (e.g., assessing personality style), should be considered.

Finally, behavioral and neurobiological similarities have been found between AN and common comorbid conditions, such as cluster C personality disorder and autism spectrum disorder [28, 29]. Considering personality style might give us a better understanding of the shared social deficits and behavioral rigidity within these groups, and possibly also help us understand at least some of the poor treatment outcomes for both AN and autism [30, 31].

### Limitations

The study has several limitations. First, conclusions cannot be drawn about causality, since the design is cross-sectional. Some studies have shown that traits like obsessive-compulsiveness and perfectionism predict the risk of developing EDs [1, 32]. Others have shown that starvation is a state which alters an individual’s thoughts, feelings, and behaviors, which also may affect the personality assessment [33]. However, rigid behaviors seem to persist even after recovery from AN with weight restoration [34]. This supports the idea of overcontrol as, at least partially, a personality style rather than a consequence of starvation. Second, even though our sample was large enough to detect meaningful differences in most pairwise comparisons, it was probably insufficient to detect smaller, yet clinically meaningful differences. However, sample size in our study is larger than many other studies investigating personality style in EDs [4, 6, 23]. Also, effect sizes provide a valid indication of the strength of the relation, beyond the issue of power and significance. Third, some of the participants in the comparison groups might also suffer from EDs, which was not assessed in this study. Levels of EDs among those with BPD have been reported to be as high as 50% [35], limiting the precision in our findings. However, excluding these individuals would make the group less representative of the actual clinical BPD group, and it is important to remember that BPD is their main diagnosis. Also, while the prevalence of EDs in BPD is high, the prevalence of BPD among those with EDs is lower: around 2% in ANr, 16% in ANbp, and 26% in BN [1, 36]. Fourth, recoding might call into question the validity of the DSM-5 diagnoses. However, due to the concrete parameters used for recoding (weight, amenorrhea, and instances of binge eating/compensation), the potential errors due to this recoding are considered to be minimal. Finally, the thirteen items included in the EUC-13 were in some cases extracted from the full version of the EUC administered at the beginning of the study. This could have some impact on how those participants rated the items. However, additional analyses showed that there were no significant differences in outcome regardless of the version of the EUC, reducing the risk for measurement error.

### Conclusion and future research

Classified according to current diagnostic criteria (DSM-5), overcontrol is higher in ANr and ANN than in BN, NC, and BPD. In addition, AAN show clear similarities with ANr in terms of overcontrol. Taking personality styles into account may improve our understanding of certain characteristics in EDs, such as social deficits and rigidity that are attributed to poor treatment outcome. Future research should evaluate the stability of these personality styles after remission from ED, and evaluate the presence of these styles in a larger variety of ED groups such as BED and subclinical BN.

## Data Availability

Data will not be made publicly available due to confidentiality, but can be made available upon reasonable request to the corresponding author.

## Abbreviations

AAN: Atypical anorexia nervosa
AN: Anorexia nervosa
ANbp: Anorexia nervosa binge eating/purging subtype
ANr: Anorexia nervosa restricting subtype
BED: Binge eating disorder
BMI: Body mass index
BN: Bulimia nervosa
BPD: Borderline personality disorder
DSM: Diagnostic and statistical manual of mental disorders
ED: Eating disorder
EDE: Eating disorder examination
ER: Ego resilience
EUC: Ego undercontrol
GLZM: Generalized linear model
NC: Non-clinical
PABAK: Prevalence and bias adjusted kappa
SCID: Structured clinical interview
SE: Standard error
